# Inter- and intra-examiner reliability of short-term measurement of heart rate variability on rest in patients hospitalized with COVID-19

**DOI:** 10.1038/s41598-024-77558-5

**Published:** 2024-11-04

**Authors:** Aldair Darlan Santos-de-Araújo, Murilo Rezende Oliveira, André Pontes-Silva, Laise Nunes Rodrigues, Cyrene Piazera Silva Costa, Renan Shida Marinho, Sigrid de Sousa dos Santos, Ross Arena, Shane A. Phillips, Daniela Bassi-Dibai, Audrey Borghi-Silva

**Affiliations:** 1 Physical Therapy Departarment, Universidade Federal de São Carlos, São Carlos, SP Brazil; 2 Department of Dentistry, Universidade CEUMA, São Luís, MA Brazil; 3 Medicine Departarment, Universidade Federal de São Carlos, São Carlos, SP Brazil; 4 Department of Physical Therapy, College of Applied Health Sciences, University of Illinois, Chicago, Chicago, USA; 5 Postgraduate Program in Management in Health Programs and Services, Universidade CEUMA, São Luís, MA Brazil; 6 Cardiopulmonary Physiotherapy Laboratory, Physical Therapy Departament, Universidade Federal de São Carlos, Rodovia Washington Luiz, Postal Code - 13565- 905, São Carlos, SP Brazil

**Keywords:** Heart rate variability, COVID-19, Hospitalization, Reliability, Cardiovascular biology, Data processing

## Abstract

Measures reflecting cardiac sympathovagal activity, particularly those associated with heart rate variability (HRV), are widely recognized and utilized in both scientific and clinical contexts. This study aimed to assess the inter- and intra-examiner reliability of short-term HRV parameters in patients hospitalized with coronavirus disease 2019 (COVID-19). A total of 103 patients (both sexes) diagnosed with COVID-19 were included in the study. HRV was analyzed using both linear and nonlinear methods. Reliability was evaluated through intraclass correlation coefficient (ICC_2.1_), minimum detectable change (MDC), standard error of measurement (SEM), and coefficient of variation (CV). According to Fleiss’ criteria, excellent reliability was demonstrated, with ICC values ranging from 0.970 to 0.999 for Examiner 1, and from 0.956 to 0.999, for Examiner 2. In the inter-examiner analysis, the ICCs of HRV parameters ranged from 0.972 to 0.999. SEM values for intra-examiner reliability for Examiner 1 ranged from 0.02 to 5.64, with MDC values from 0.05 to 15.64, and CV (%) from 0.28 to 8.04. For Examiner 2, SEM values ranged from 0.02 to 8.18, MDC values from 0.05 to 22.68, and CV (%) from 0.24 to 8.14. For inter-examiner reliability, SEM values ranged from 0.02 to 6.17, MDC from 0.06 to 17.11, and CV (%) from 0.34 to 9.81. Across all analyses, CVs for HRV parameters remained below 10%. Considering different time points and different examiners, short-term resting HRV measurements in patients hospitalized with COVID-19, as evaluated using a portable heart rate device, exhibit high reliability.

## Introduction

Although initial evidence primarily focused on elucidating respiratory system impairments caused by the tropism of severe acute respiratory syndrome coronavirus 2 (SARS-CoV-2) to lung parenchyma^1–3^, the multisystemic nature of the disease soon became the subject of extensive research. This broader perspective highlighted significant effects on other critical systems, such as the nervous^4^ and cardiovascular systems^5,6^. In particularr, cardiac autonomic dysfunction, which has been observed in individuals infected with coronavirus disease 2019 (COVID-19)^7^, even in the early stages, is often associated with poor clinical outcomes^8^.

Inflammatory responses linked to COVID-19 affect the vagus nerve, which plays a key role not only as an inflammatory neuroimmunomodulator^9^ but also in cardiac autonomic regulation, providing valuable insights into cardiovascular health through its impact on sinoatrial node function^10^. Several analytical methods can be employed to assess cardiac autonomic function^11^. Among them, heart rate variability (HRV) has been considered an attractive approach as a non-invasive, simple measure^11^ with diagnostic and prognostic potential. HRV has been shown predict hospitalization duration^12,13^, mortality^14,15^ and can guide therapeutic interventions^16^.

Despite these promising findings, the role of HRV in COVID-19 patients remains inconclusive. The evidences so far indicate the existence of a sympatho-vagal imbalance and the variability in results may stem from the use of different tools (such as conventional electrocardiogram and portable devices), analysis methods (short- versus long-term assessments), and data processing techniques (varying software)^17^. Initial research in patients hospitalized with COVID-19 suggests the clinical utility of short-term HRV measurements^13,15,18^. However, only one study has explored intra- and inter-rater reliability in mild, non-hospitalized post-COVID patients^19^ and none have investigated the reliability of HRV analyses in patients with moderate to severe COVID-19 during hospitalization considering inter- and intra-examiner reliability and the differences attributed to measurement errors, which are crucial for ensuring consistency in data collection and analysis^20^.

Measures reflecting cardiac sympathovagal activity, particularly HRV, are widely recognized and applied in both scientific and clinical contexts^11^. However, HRV measurements are susceptible to bias^21,22^ and the progress in understanding this outcome depends on the intimate relationship between the reliability of the measurements protocols, their discriminative ability, and scientific reproducibility^11^. Clinically, this can help avoid analysis biases, standardize data collection and processing, and clarify he magnitude of variations assessments conducted by the same or different examiners. Given the susceptibility of HRV to various biases, ranging from data collection to analysis, this study aimed to investigate the inter- and intra-examiner reliability of HRV parameters in patients hospitalized with COVID-19. We hypothesize that this analysis will demonstrate acceptable reliability indices when assessed by the same examiner or by independent examiners.

## Methodology

### Study design

This reliability study was conducted in accordance with the Guidelines for Reporting Reliability and Agreement Studies (GRRAS)^23^. The investigation at the University Hospital (UH) of the Universidade Federal de São Carlos (São Paulo, Brazil), Santa Casa de Misericórdia Hospital of São Carlos (São Paulo, Brazil) (protocol number: 4120437) and Universidad Ceuma (São Luís, MA, Brazil) (protocol number: 4.610.070), following approval by the Research Ethics Committee of the involved institutions and in accordance with the Declaration of Helsinki. All participants or their legal representatives/familiar members were informed about the study’s purpose, and informed consent was obtained directly from the patient when possible. In cases where the patient could not provide formal consent, it was obtained from a legal representative or family member.

### Sample size

According to by Eliasziw and Donner^23^, considering a minimum acceptable ICC value of 0.40 and an expected value of 0.75, along with a sample loss estimate of 15%, 80% statistical power, and a 5% α error, the minimum number of participants required for the current study was calculated to be^30^.

### Participants

This study included patients of both sexes, aged ≥ 18 years, admitted to a hospital setting (Intensive Care Unit or ward) within 24–48 h of admission to the University Hospital of São Carlos, Santa Casa de Misericordia Hospital of São Carlos (São Carlos, SP, Brazil), and High Complexity Hospital Dr. Carlos Macieira (São Luís, MA, Brazil). All participants were breathing spontaneously on room air and had a confirmed positive diagnosis for COVID-19 via real-time reverse transcriptase polymerase chain reaction (RT-PCR) using nasopharyngeal swabs, between July 2020 and December 2021. Data collection occurred between until 48 h after hospitalization. Three researchers were responsible for collecting the biological signals for analysis. The two researchers based São Carlos, SP, conducted the data collection at both hospitals based on patient demand, while the researcher based in São Luís, MA, was responsible for collecting data at the respective hospital city’s hospital. All researchers had previous experience with this protocol, and data collection followed standardized guidelines and evidence^22,24^.

Patients were categorized by disease severity^25^, with those presenting symptoms such as fever, cough, sore throat, malaise, headache, muscle pain, nausea, vomiting, diarrhea, anosmia or dysgeusia but without shortness of breath or abnormal chest imaging classified as having COVID-19 flu-like syndrome. Those with clinical symptoms or radiological evidence of lower respiratory tract disease and oxygen saturation (SpO_2_) ≤ 94% on room air were classified as Moderate COVID-19. Patients with SpO_2_ ≤ 93% on room air, an arterial partial pressure of oxygen to fraction of inspired oxygen ratio (PaO_2_/FiO_2_) < 300, or marked tachypnea with a respiratory rate > 30 breaths/min or pulmonary infiltrates > 50%, were classified as having Severe COVID-19^26^.

The following exclusion criteria were applied: (1) patients or family members who did not consent to participate in the study by signing the Informed Consent Form; (2) those receiving palliative care, as hospital regulations precluded assessment of these patients; (3) cases of rehospitalization due to COVID-19 to avoid bias related to chronic infection effects; (4) patients using non-invasive (NIV) and invasive mechanical ventilation (IMV) during evaluation, as these can affect vascular function and autonomic nervous system responses^27^; (5) patients in the prone position; (6) patients with cardiac arrhythmias, pacemakers, or atrioventricular block; (7) use of vasoactive drugs; (8) neoplasms; (9) declared illicit drugs users; and (10) pregnancy.

### Clinical and sociodemographic characteristics

Clinical and sociodemographic data were collected from medical records, including age (years), gender (female and male), weight in kilograms (kg), height in meter (m), disease severity, presence of comorbidities, medication use, and need for oxygen supplementation. Body mass index (BMI) was calculated using the ratio of weight (kg) to height squared (m²)^28^. BMI categories were defined as follows: normal weight (18.5–24.9 kg/m²), overweight (25.0–29.9 kg/m²), obesity class I (30.0–34.9 kg/m²), obesity class II (35.0–39.9 kg/m²), and obesity class III (≥ 40.0 kg/m²)/ Patients were classified into categories according to previously established BMI reference values: BMI between 18.5 and 24.9 kg/m² were considered as normal weight; between 25.0 and 29.9 kg/m², classified as overweight; between 30.0 and 34.9 kg/m², obesity class I; between 35.0 and 39.9 kg/m², obesity class II; and those with BMI ≥ 40.0 kg/m² were included in the obesity class III^28^.

### Heart rate variability assessment

In the morning, before data collection, patients were asked about their self-reported sleep quality of the previous night, whether they had consumed caffeine, and/or engaged in moderate to vigorous exercise with the rehabilitation team within the previous 12 h. If any of these conditions were present, the patient was excluded from evaluation^22^. Evaluations were conducted in the morning to minimize circadian rhythm effects. Additionally, patients were positioned in supine position, in a temperature-controlled environment (22–24 °C), relative humidity within 50–60%, and encouraged to relax, breathe spontaneously, refrain from speaking or moving, and avoid sleeping during data collection. Before the evaluation, patients remained at rest for 10 min to stabilize their heart rate, after which data collection commenced^22^. A Polar H10 chest strap monitor (Polar Electro Inc., Bethpage, NY, USA) was placed bellow the patient’s nipples, ensuring the sensor was in direct contact with the skin and securely fitted. The center of the sensor was aligned to the sternum, and the strap was adjusted to prevent movement without causing discomfort. Heart rate intervals (iR-R) were recorded for 10 min and the data collected was transferred via Bluetooth to an email address for later processing and analysis.

### Data processing and analysis

Data processing followed the method described by Santos-de-Araújo et al.^29^ HRV data were analyzed using Kubios HRV software (University of Eastern Finland), which offers comprehensive options for linear (time and frequency domain) and nonlinear analysis^21,24^. HRV signals were evaluated in 5-minute stable sessions^22^. Signal quality was visually assessed based on the following criteria and the section with the best stationarity was chosen: (1) absence of large outliers in the R-R intervals; (2) equidistant R-R intervals; and (3) adherence to Gaussian distribution of R-R intervals and heart rate^24,30^. The series of R-R intervals underwent trend reduction using a smoothing method with a lambda value of 500 and cubic interpolation at a standard rate of 4 Hz^24,30^. Artifacts were removed, when necessary, using the medium filter of the Kubios software as it does not present any risk of severe interpolations that compromise the results, as previously recommended^30^.

HRV was analyzed using linear (time and frequency domain) and nonlinear mathematical and statistical models. Linear parameters in the time domain included the mean R-R intervals in milliseconds (ms) (Mean R-R), standard deviation of all intervals between two consecutive normal heartbeats in ms (SDNN), mean heart rate (beats per minute) (Mean HR), root mean square of successive RR interval differences (RMSSD), triangular index (RR Tri), and triangular interpolation of the R-R interval histogram (TINN). I the frequency domain, the variables were extracted using the normalized powers method: low frequency band (0.04–0.15 Hz) (LF) and high frequency band (0.15–0.4 Hz) (HF) in normalized units (n.u.). In the non-linear indices, were analyzed the following variables: Poincaré plot standard deviation perpendicular the line of identity (SD1), Poincaré plot standard deviation along the line of identity (SD2), ratio of SD2 to SD1 (SD2/SD1), detrended fluctuation analysis, which describes short-term fluctuations (DFAα 1), detrended fluctuation analysis, which describes long-term fluctuations (DFAα 2), approximate entropy (Apen), Sample entropy (SampEn)^21,31^.

### Statistical analysis

Quantitative data were presented as mean ± standard deviation or median and minimum and maximum value, while qualitative data were reported as frequencies and percentages. Normality was assessed using the Kolmogorov-Smirnov test. Paired t-test were used for intra- and inter-examiner comparisons when normality assumptions were met; otherwise, the Mann-Whitney test was applied. A p-value < 0.05 was considered statistically significant.

Intraclass correlation coefficient (ICC_2,1_)^32^, minimum detectable change (MDC), standard error of measurement (SEM), coefficient of variation (CV) and Bland–Altman analysis (mean difference [bias] and limits of agreement) were used to assess inter- and intra-examiner reliability and reproducibility of HRV parameters. ICC values were interpreted according to Fleiss (1986)^36^: <0.40 as low; 0.40–0.75 as moderate; 0.75–0.90 as substantial; and > 0.90 as excellent reliability. MDC, SEM and CV were included to complement the interpretation of measurement error. MDC was calculated as 1.96×SEM×√2^37^ while SEM was calculated as standard deviation X √(1-ICC)^34^. CV was calculated as the ratio of the standard deviation to the mean value: and represents the extent of variability of an assay: the greater the CV, the greater the error in the assay and can be calculated using the formula CV (%) = (standard deviation)/mean) X 100^38^. Bland-Altman analysis was performed to assess agreement between the two quantitative measurements, allowing the visualization of differences and the identification of possible biases in the intra- and inter-examiner analyses^35^. All analyzes were performed using the Statistical Package for the Social Sciences software (SPSS version 20, SPSS Inc., Chicago, IL, USA).

## Results

**Fig. 1:**
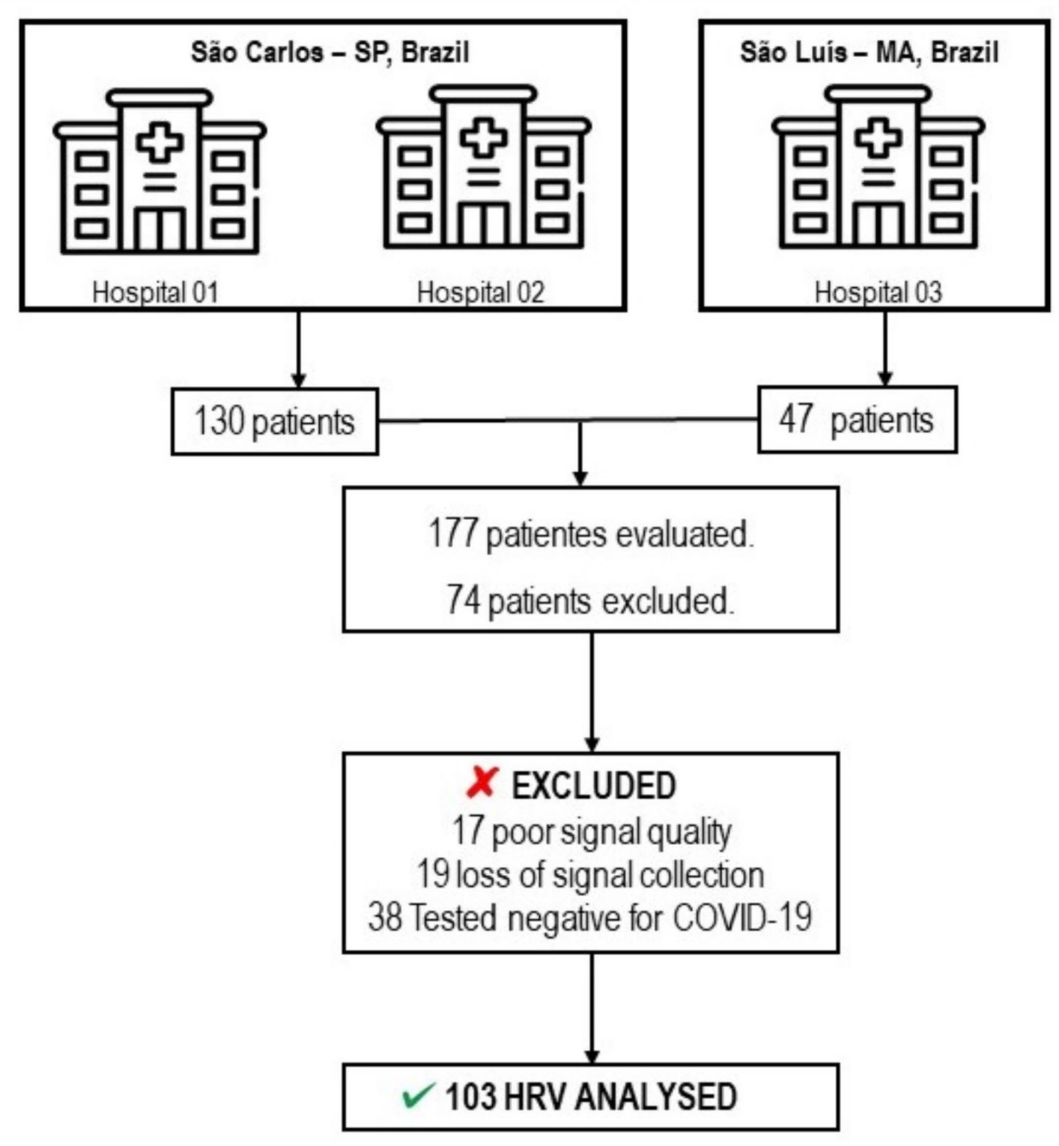
Flowchart of patient screening and study design.

Initially, 177 patients were evaluated; however, 74 were excluded for the following reasons: 17 had poor signal quality, 19 experienced signal collection loss, and 38 tested negative for COVID-19. Thus, a total of 103 patients were included in this study. Figure 1 presents the flowchart illustrating the study design and included patients. The characteristics of the sample are detailed in Table 1. The majority of the sample were male (57%), overweight (47%), admitted to the ward (80%), requiring oxygen supplementation (66%), ex-smokers (53%), and sedentary (79%). In terms of disease severity, most patients were classified as moderate (*n* = 95, 92%). The most prevalent comorbidities were hypertension (58%), smoking (45%), and type 2 diabetes mellitus (35%). The most frequently used medication classes included: (1) antibiotics (64%), (2) anticoagulants (33%), and (3) analgesics (33%).

**Table 1:** General characteristics of the study sample (*n* = 103).

Variables	Mean (SD) or *n* (%)
Age (years)	61 ± 16
Gender	
Male, n (%)	59 (57)
Female, n (%)	44 (43)
Mass (kg)	81.33 ± 16.84
Height (m)	1.68 ± 0.09
BMI (kg/m²)	28.59 ± 4.76
Normal	23 (22)
Overweight	48 (47)
Obese class I	23 (22)
Obese class II	5 (5)
Obese class III	4 (4)
Severity	
Moderate	95 (92)
Severe	8 (8)
Evaluation location, n (%)	
Ward	82 (80)
Intensive Care Unit	21 (20)
Oxygen supplementation, n (%)	68 (66)
Interface, n (%)	
Nasal catheter	56 (54)
Venturi mask	12 (9)
Comorbidities, n (%)	
Hypertension	60 (58)
Obesity	13 (13)
Type 2 Diabetes	36 (35)
COPD	7 (7)
Current Smokers	46 (45)
Ex-smokers	54 (53)
Non smokers	3 (3)
Sedentarism	81 (79)
Medications, n (%)	
Corticosteroid	19 (18)
Anticoagulant	34 (33)
Antidiabetic agentes	24 (23)
Analgesic	34 (33)
Antihypertensive	9 (9)
Antibiotic	66 (64)
Anti-inflammatory	9 (9)
Antivirals	6 (6)

Values are mean ± standard deviation (SD) or absolute values and percentage (%); kg: kilos; m: meter; BMI: body mass index; kg/m²: kilos per square meter; COPD: chronic obstructive pulmonary disease.

**Fig. 2:**
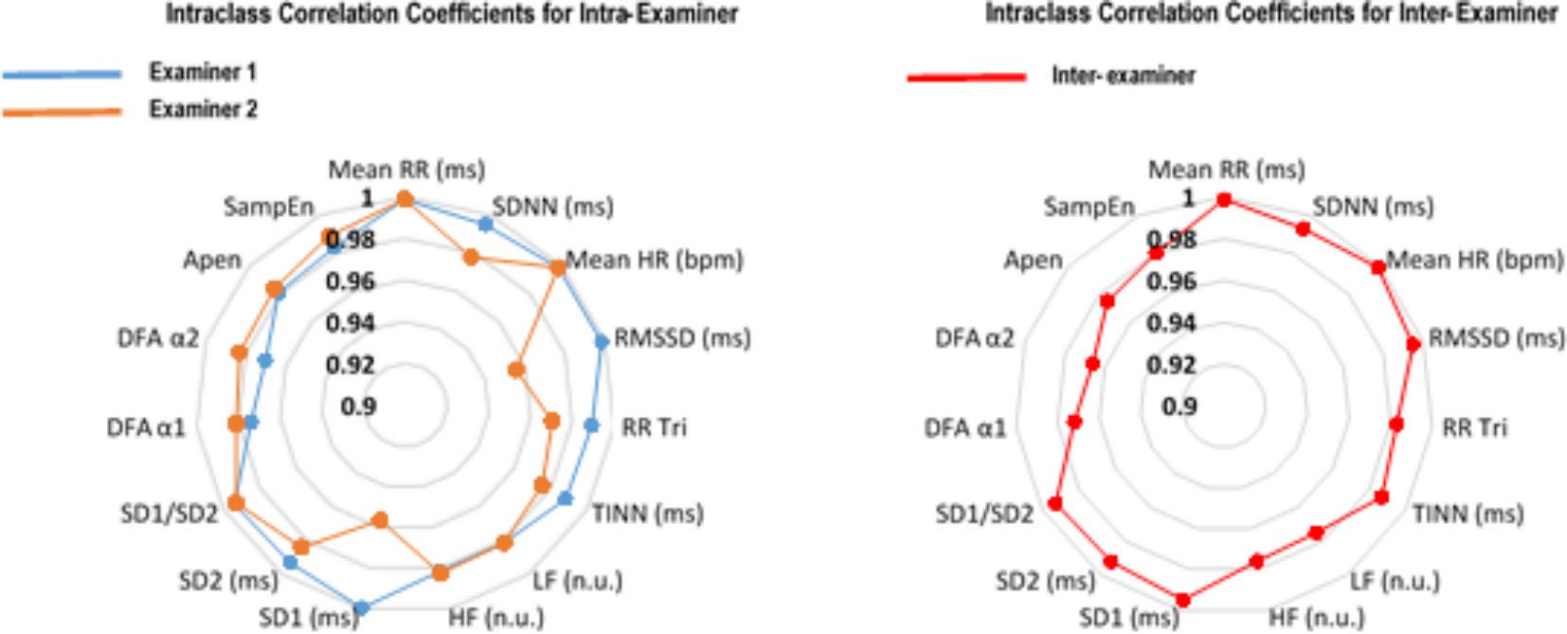
Radar chart comparing intraclass correlation coefficients by assessing intra- and inter-examiner reliability.Mean RR: average R-R interval duration between heartbeats; ms: milliseconds; SDNN: Standard deviation of RR intervals; Mean HR: average heart rate; bpm: in beats per minute; RMSSD: root mean square differences of successive RR intervals; RR Tri: integral of the RR intervals histogram divided by the height of the histogram; LF: normalized unit in the low-frequency band; HF: normalized unit in the high-frequency band; SD1: standard-deviation of the instant beat-to-beat variability; SD2: long-term standard-deviation of continuous RR intervals; DFA α1: purified trend fluctuations (short-term scale); DFA α2: purified trend fluctuations (long-term scale); ApEn: approximate entropy; SampEn: sample entropy.

Table 2 presents the mean and standard deviation of HRV indices in the supine position, analyzed by both Examiner 1 and Examiner 2. No statistically significant differences were observed in intra- and inter-examiner comparative analyses (*p* > 0.05). Figure 2 shows a radar chart with intra- and inter-examiner ICC values. According to Fleiss’ interpretation, excellent reliability was found in the ICC values, which ranged from 0.970 to 0.999 for Examiner 1, and from 0.956 to 0.999 for Examiner 2. The inter-examiner analysis also demonstrated excellent reliability, with ICC values for HRV parameters ranging from 0.972 to 0.999, according to Fleiss’ interpretation. Table 3 shows the intra-examiner reliability results for Examiner 1, with SEM values ranging from 0.02 to 5.64, MDC from 0.05 to 15.64 and CV (%) from 0.28 to 8.04. Similarly, Table 4 presents the intra-examiner reliability results for Examiner 2, with SEM values ranging from 0.02 to 8.18, MDC from 0.05 to 22.68, and CV (%) from 0.24 to 8.14. Table 5 provides inter-examiner results, with SEM values ranging from 0.02 to 6.17, MDC from 0.06 to 10.01, and CV (%) from 0.34 to 9.81. Overall, CVs for HRV parameters were less than 10% in all analyzes (both intra- and inter-examiner).

**Table 2:** Mean values (standard deviation) of heart rate variability (HRV) in individuals with COVID-19 hospitalized in the supine position.

HRV indices	Examiner 1	Examiner 1	*P* value	Examiner 2	Examiner 2	*P* value	*P* value Examiner 1 x Examiner 2 (Test)	*P* value Examiner 1 x Examiner 2 (Retest)
	Test	Retest		Test	Retest			
Time domain								
Mean RR (ms)	736.50 ± 130.50	736.77 ± 130.08	0.620	736.69 ± 130.75	736. 49 ± 130.77	0.605	0.735	0.600
SDNN (ms)	17.44 ± 10.92	17.45 ± 10.87	0.927	17.20 ± 10.84	16.97 ± 10.35	0.445	0.193	0.092
Mean HR (bpm)	84.23 ± 16.96	84.19 ± 16.99	0.562	84.22 ± 17.04	84.27 ± 17.21	0.437	0.938	0.295
RMSSD (ms)	15.54 ± 10.55	15.60 ± 10.58	0.479	15.31 ± 10.57	14.90 ± 9.76	0.315	0.112	0.084
RR Tri	4.94 ± 2.75	4.98 ± 2.78	0.414	4.89 ± 2.70	4.95 ± 2.73	0.466	0.414	0.719
TINN	89.66 ± 54.59	88.55 ± 52.99	0.323	87.33 ± 53.68	86.16 ± 51.96	0.470	0.058	0.073
Frequency domain								
LF (n.u.)	66.02 ± 21.91	65.77 ± 22.75	0.682	65.55 ± 22.25	66.96 ± 22.16	0.116	0.491	0.120
HF (n.u.)	33.86 ± 21.88	34.10 ± 22.72	0.692	34.32 ± 22.19	32.91 ± 22.14	0.115	0.488	0.124
Nonlinear analysis								
SD1 (ms)	11.00 ± 7.47	11.04 ± 7.50	0.472	10.84 ± 7.48	10.55 ± 6.91	0.316	0.113	0.085
SD2 (ms)	21.63 ± 14.21	21.62 ± 14.15	0.987	21.31 ± 14.08	21.10 ± 13.61	0.537	0.200	0.094
SD2/SD1	2.20 ± 1.03	2.19 ± 1.04	0.582	2.21 ± 1.05	2.23 ± 1.07	0.200	0.841	0.112
Apen	1.17 ± 0.13	1.17 ± 0.12	0.616	1.17 ± 0.13	1.17 ± 0.13	0.544	0.459	0.765
SampEn	1.63 ± 0.36	1.65 ± 0.37	0.137	1.65 ± 0.36	1.65 ± 0.36	0.472	0.246	0.749
DFA α1	1.12 ± 0.35	1.12 ± 0.35	0.725	1.13 ± 0.36	1.13 ± 0.37	0.844	0.828	0.423
DFA α2	0.56 ± 0.21	0.57 ± 0.20	0.600	0.56 ± 0.19	0.56 ± 0.20	0.496	0.882	0.826

Mean RR: average R-R interval duration between heartbeats; ms: milliseconds; SDNN: Standard deviation of RR intervals; Mean HR: average heart rate; bpm: in beats per minute; RMSSD: root mean square differences of successive RR intervals; RR Tri: integral of the RR intervals histogram divided by the height of the histogram; LF: normalized unit in the low-frequency band; HF: normalized unit in the high-frequency band; SD1: standard-deviation of the instant beat-to-beat variability; SD2: long-term standard-deviation of continuous RR intervals; DFA α1: purified trend fluctuations (short-term scale); DFA α2: purified trend fluctuations (long-term scale); ApEn: approximate entropy; SampEn: sample entropy. No significant differences were found intra or inter examinator (*p* > 0.05).

**Table 3:** Intrarater reliability HRV analysis of patients with COVID-19 hospitalized in the supine position (Examiner 01).

HRV Index	ICC	SEM	SEM (%)	MDC	MDC (%)	Bias ± SD	CV (%)	95% limit of agreement
Time domain								
Mean RR (ms)	0.999	4.12	0.56	11.42	1.55	-0.26 ± 5.37	0.28	-10.79, 10.26
SDNN	0.995	0.77	4.42	2.14	12.24	-0.01 ± 1.58	3.15	-3.12, 3.09
Mean HR (bpm)	0.999	0.54	0.64	1.49	1.77	0.03 ± 0.60	0.28	-1.14, 1.21
RMSSD (ms)	0.999	0.33	2.15	0.93	5.95	-0.05 ± 0.81	2.25	-1.65, 1.54
RR Tri	0.990	0.28	5.57	0.77	15.45	-0.04 ± 0.54	3.83	-1.11, 1.03
TINN	0.989	5.64	6.33	15.64	17.55	1.10 ± 11.31	4.25	-21.06, 23.28
Frequency domain								
LF (n.u.)	0.981	3.08	4.67	8.53	12.85	0.25 ± 6.12	4.30	-11.75, 12.24
HF (n.u.)	0.981	3.07	9.05	8.52	25.07	-0.23 ± 6.12	8.04	-12.24, 11.76
Nonlinear analysis								
SD1 (ms)	0.999	0.24	2.15	0.66	5.95	-0.04 ± 0.57	2.25	-1.17, 1.09
SD2 (ms)	0.993	1.19	5.49	3.29	15.21	0.01 ± 2.31	3.71	-4.53, 4.54
SD1/SD2	0.994	0.08	3.65	0.22	10.12	0.01 ± 0.15	2.84	-0.29, 0.31
DFA α1	0.974	0.06	5.04	0.16	13.97	0.01 ± 0.11	5.43	-0.21, 0.22
DFA α2	0.970	0.04	6.28	0.10	17.42	-0.01 ± 0.07	5.63	-0.14, 0.14
Apen	0.981	0.02	1.47	0.05	4.08	-0.01 ± 0.03	1.33	-0.07, 0.06
SampEn	0.983	0.05	2.90	0.13	8.04	-0.01 ± 0.09	2.66	-0.19, 0.17

HRV: heart rate variability; SEM: standard error of measurement; MDC: minimal detectable change; Mean RR: average R-R interval duration between heartbeats; ms: milliseconds; SDNN: Standard deviation of RR intervals; Mean HR: average heart rate; bpm: in beats per minute; RMSSD: root mean square differences of successive RR intervals; RR Tri: integral of the RR intervals histogram divided by the height of the histogram; LF: normalized unit in the low-frequency band; HF: normalized unit in the high-frequency band; SD1: standard-deviation of the instant beat-to-beat variability; SD2: long-term standard-deviation of continuous RR intervals; DFA α1: purified trend fluctuations (short-term scale); DFA α2: purified trend fluctuations (long-term scale); ApEn: approximate entropy; SampEn: sample entropy.

**Table 4:** Intrarater reliability HRV analysis of patients with COVID-19 hospitalized in the supine position (Examiner 02).

HRV Index	ICC	SEM	SEM (%)	MDC	MDC (%)	Bias ± SD	CV (%)	95% limit of agreement
Time domain								
Mean RR (ms)	0.999	4.13	0.56	11.46	1.56	0.20 ± 3.92	0.24	-7.48, 7.88
SDNN	0.978	1.57	9.20	4.36	25.50	0.23 ± 3.09	3.09	-5.82, 6.30
Mean HR (bpm)	0.999	0.54	0.64	1.50	1.78	-0.04 ± 0.58	0.24	-1.18, 1.10
RMSSD (ms)	0.956	2.13	14.12	5.91	19.13	0.41 ± 4.16	3.16	-7.74, 8.56
RR Tri	0.971	0.46	9.40	1.28	26.05	-0.06 ± 0.91	4.47	-1.85, 1.72
TINN	0.976	8.18	9.43	22.68	26.15	1.16 ± 16.31	3.61	-30.79, 33.12
Frequency domain								
LF (n.u.)	0.981	3.06	4.62	8.48	12.81	-1.41 ± 5.86	4.50	-12.91, 10.08
HF (n.u.)	0.982	2.97	8.85	8.24	24.52	1.40 ± 5.79	8.14	-9.94 ± 12.76
Nonlinear analysis								
SD1 (ms)	0.956	1.51	14.11	4.18	39.12	0.29 ± 2.94	3.16	-5.48, 6.06
SD2 (ms)	0.984	1.75	8.26	4.85	22.89	0.21 ± 3.47	3.36	-6.60, 7.03
SD1/SD2	0.993	0.09	3.99	0.25	11.07	-0.02 ± 0.17	2.83	-0.36, 0.31
DFA α1	0.981	0.05	4.45	0.14	12.34	-0.01 ± 0.10	4.91	-0.20, 0.19
DFA α2	0.983	0.03	4.54	0.07	12.58	-0.01 ± 0.05	4.72	-0.10, 0.09
Apen	0.984	0.02	1.41	0.05	3.90	0.01 ± 0.03	1.17	-0.06, 0.06
SampEn	0.989	0.04	2.29	0.10	6.34	-0.01 ± 0.07	2.08	-0.15, 0.14

HRV: heart rate variability; SEM: standard error of measurement; MDC: minimal detectable change; Mean RR: average R-R interval duration between heartbeats; ms: milliseconds; SDNN: Standard deviation of RR intervals; Mean HR: average heart rate; bpm: in beats per minute; RMSSD: root mean square differences of successive RR intervals; RR Tri: integral of the RR intervals histogram divided by the height of the histogram; LF: normalized unit in the low-frequency band; HF: normalized unit in the high-frequency band; SD1: standard-deviation of the instant beat-to-beat variability; SD2: long-term standard-deviation of continuous RR intervals; DFA α1: purified trend fluctuations (short-term scale); DFA α2: purified trend fluctuations (long-term scale); ApEn: approximate entropy; SampEn: sample entropy.

**Table 5:** Interrater reliability HRV analysis of patients with COVID-19 hospitalized in the supine position.

HRV Index	ICC	SEM	SEM (%)	MDC	MDC (%)	Bias ± SD	CV (%)	95% limit of agreement
Time domain								
Mean RR (ms)	0.999	4.13	0.56	11.45	1.55	0.19 ± 5.83	0.34	-11.24, 11.63
SDNN	0.993	1.61	9.32	4.47	25.83	-0.23 ± 1.83	4.58	-3.82, 3.35
Mean HR (bpm)	0.999	0.54	0.64	1.49	1.77	-0.01 ± 0.69	0.34	-1.36, 1.35
RMSSD (ms)	0.995	0.75	4.84	2.07	13.42	-0.23 ± 1.47	3.76	-3.12, 2.66
RR Tri	0.983	0.36	7.23	0.98	20.04	-0.05 ± 0.70	5.33	-1.44, 1.33
TINN	0.987	6.17	6.97	17.11	19.33	-2.33 ± 12.32	5.76	-26.47, 21.81
Frequency domain								
LF (n.u.)	0.975	3.49	5.31	9.68	14.71	-0.47 ± 6.89	5.90	-13.98, 13.04
HF (n.u.)	0.976	3.41	10.01	9.46	27.76	0.46 ± 6.81	9.81	-12.88, 13.81
Nonlinear analysis								
SD1 (ms)	0.995	0.53	4.85	1.47	13.43	-0.16 ± 1.04	3.76	-2.21, 1.88
SD2 (ms)	0.992	1.27	5.89	3.51	16.33	-0.32 ± 2.52	5.16	-5.25, 4.61
SD1/SD2	0.993	0.09	3.95	0.24	10.94	0.01 ± 0.17	3.82	-0.33, 0.34
DFA α1	0.972	0.06	5.30	0.16	14.70	0.01 ± 0.11	5.99	-0.22, 0.23
DFA α2	0.966	0.04	6.69	0.10	18.54	-0.01 ± 0.07	6.58	-0.14, 0.14
Apen	0.975	0.02	1.76	0.06	4.87	0.01 ± 0.04	1.67	-0.07, 0.08
SampEn	0.980	0.03	5.05	0.08	14.00	0.01 ± 0.10	3.16	-0.18, 0.21

HRV: heart rate variability; SEM: standard error of measurement; MDC: minimal detectable change; Mean RR: average R-R interval duration between heartbeats; ms: milliseconds; SDNN: Standard deviation of RR intervals; Mean HR: average heart rate; bpm: in beats per minute; RMSSD: root mean square differences of successive RR intervals; RR Tri: integral of the RR intervals histogram divided by the height of the histogram; LF: normalized unit in the low-frequency band; HF: normalized unit in the high-frequency band; SD1: standard-deviation of the instant beat-to-beat variability; SD2: long-term standard-deviation of continuous RR intervals; DFA α1: purified trend fluctuations (short-term scale); DFA α2: purified trend fluctuations (long-term scale); ApEn: approximate entropy; SampEn: sample entropy.

## Discussion

The findings of the current study are: (1) intra- and inter-rater analysis of short-term HRV assessed in patients hospitalized with COVID-19 is a reliable measure; and (2) the coefficients of variation in both analyzes presented values lower than 10%. The main contribution of this investigation is to assure professionals that the analysis of heart rate variability (HRV) in hospitalized COVID-19 patients is reliable, provided it follows the standards established in the literature^22,24^. Furthermore, despite the sample loss, the results demonstrate that HRV can be used as a clinical outcome in a hospital setting, as most of the data collection showed good signal quality. Although the focus of this study was not specifically to investigate the HRV behavior in this population, the results provide valuable insight into cardiac autonomic modulation for professionals dealing with these patients. The prognostic power linked to HRV, which indirectly reflects the activity of the autonomic nervous system, has been the subject of several scientific investigations in different populations^36,29^. However, biases that can compromise signal quality and result interpretation have raised concerns about its clinical applicability. This underscores the importance of standardizing collections, analyzes (methods used, intra- and inter-examiner differences), and the identification of artifacts-whether technical (individual movements, poorly fixed electrode) or physiological origin (ectopic beats)^37^.

In patients infected with COVID-19 at both outpatient and hospital levels, previous investigations have assessed HRV as a key outcome across various levels of clinical severity^7,8,13,18^. For patients who did not require hospitalization, HRV analysis demonstrated good intra- and inter-examiner reliability rates, supporting its use clinically and scientifically^19^. Interestingly, some studies have also presented results from HRV evaluation in hospitalized COVID-19 patients^8,13,38^. Although collection in such environments is feasible and encouraged, a systematic review found significant methodological differences between studies that hindered direct comparisons^39^. Therefore, methodological biases must be controlled and reported to ensure scientific reproducibility and minimize biases, particularly those that create artifacts during biological signal collection^22,39^.

Approximately 20% of our initial sample was excluded due to signal quality issues or signal loss during collection. Several aspects can justify this methodological fragility: signal quality can be compromised by factors such as movements during collection, coughing, sneezing, and even napping^22^. In this population, where coughing is a common symptom, it is difficult to control these/ factors, which may generate introduce into the data^40^. Although it is recommended to remove compromised segments from beat-to-beat data using specific filters or algorithms, doing so inappropriately or using overly restrictive filters (e.g., strong and very strong filters in Kubios software) can affect spectral components and lead to misleading results^22,30,41^. It is also important to note that the, as this device operates via Bluetooth technology, according to the manufacturer, excessive distance between the sensor and the receiving device can prevent the signal from being captured, leading to data loss. Other factors include low battery in the device attached to the patient’s chest, excessive patient movement, damaged electrodes, clothing interference, and pairing issues with device pairing.

When comparing our results with the current literature, we can note that Mol et al.^15^, found that HRV can predict mortality and referral to intensive care in hospitalized COVID-19 patients. It appears that patients with an SDNN and RMSSD < 8 ms had higher risk of intensive care unit admission, regardless of age and associated risk factors. In patients over 70, an SDNN ≥ 8 ms predicted a 3.1 times higher chance of survival compared to lower HRV. Both variables are clinically important, especially when considering that RMSSD is a sensitive measure of parasympathetic nervous system activity and the primary time-domain measure used to estimate vagally mediated changes in HRV, whereas SDNN is influenced by both nervous systems (sympathetic and parasympatheticshort) but in short-term resting recordings, the primary source of ANS variation in this variable is mediated by the parasympathetic ANS^21^. Despite the study’s limitations, when applying these cutoff points to our results, we observed that the mean differences in intra- and inter-examiner analyses are not clinically relevant. However, the limits of agreement for Examiner 2’s RMSSD variable may hold clinical relevance, as the 95% confidence interval exceeds the previously established cutoff point.

Few studies have investigated the intra- and inter-examiner reliability of HRV, and good reliability rates have been found in diabetic individuals^36^, adolescents^42^, children^43^, young adults^43^, middle-aged adults^43^ and post-COVID patients^19^, all using portable heart rate monitors for data collection and Kubios software for signal processing. Our results showed that inter-examiner reproducibility had a higher percentage of coefficient of variation in some variables, and lower ICC values compared to intra-examiner analyses, particularly in frequency domain indices and non-linear analysis. This may be partially explained by the difficulty in obtaining Gaussian distribution of R-R intervals, which can complicate the selection of the most stationary section of the signal, especially in patients with high HRV rates^43^. In view of this, it must be considered that in both analyzes (intra- and inter-examiner) the differences found can be explained by the nature of the methodological aspects used by both evaluators and the criteria used to select the best stationary section that, although it was standardized before the analyses, it still relies on the bias of the dependent evaluator to elect the section with the greatest stationarity of the signal^43^.

There is no doubt that COVID-19 affects cardiac autonomic modulation. A growing body of evidence highlights the impact of viral infections, not only from SARS-CoV-2, but also from other microorganisms responsible for major epidemics. The use of cost-effective alternative evaluation methods in medicine has gained increasing appreciation from both clinical and scientific communities, contributing to advancements in identifying, controlling and monitoring unfavorable outcomes. In practice, HRV has provided important parameters for assessing cardiovascular health. However, the scientific community has increasingly emphasized the need to address technical limitations and biases that may compromise results, particularly those related to biological signal’s nature and the lack of standardized analyzes^44,45^.

### Clinical implications

This study provides standard error of measurement (SEM) as well as minimal detectable change (MDC) data for assessing HRV in the time domain, frequency domain, and nonlinear analysis in patients hospitalized with COVID-19. Based on SEM and MDC values, this study facilitates the evaluation of COVID-19 through the autonomic assessment of the cardiac autonomic nervous system using HRV at different times and by different evaluators.

### Strenghts and limitations

Our findings underscore strengths beyond the investigation’s originality and sample size. While reliability study guidelines highlights the appropriateness of the ICC for methodological vaidation in studies^37^, we provide additional data to enhance result interpretation, including standard error of measurement and minimum detectable change for each variable. These metrics help differentiate true changes from random measurement errors. Furthermore, Bland-Altan^38^ analysis assessed agreement between intra- and inter-examiner measurements, and a coefficient of variation was used to measure relative dispersion. On the other hand, we must acknowledge some limitations: (1) the sample predominantly consisted of patients with moderate severity; and (2) patients on mechanical ventilatory support were excluded from the analyses due to the use of sedatives and neuromuscular blockers, which would intefere with cardiac autonomic modulation analysis.

## Conclusion

When evaluated by different examiners at different times, the resting short-term HRV of patients hospitalized with COVID-19, assessed using a portable heart rate device, shows excelent reliability. Additionally, we emphasize the importance of standardizing analyzes to minimize biases s and ensure scientific reproducibility.

Mean RR: average R-R interval duration between heartbeats; ms: milliseconds; SDNN: Standard deviation of RR intervals; Mean HR: average heart rate; bpm: in beats per minute; RMSSD: root mean square differences of successive RR intervals; RR Tri: integral of the RR intervals histogram divided by the height of the histogram; LF: normalized unit in the low-frequency band; HF: normalized unit in the high-frequency band; SD1: standard-deviation of the instant beat-to-beat variability; SD2: long-term standard-deviation of continuous RR intervals; DFA α1: purified trend fluctuations (short-term scale); DFA α2: purified trend fluctuations (long-term scale); ApEn: approximate entropy; SampEn: sample entropy.

## Data Availability

The datasets used and analyzed during this study are available from the corresponding author upon reasonable request.
